# Prevalence of Visual Acuity Loss or Blindness in the US: A Bayesian Meta-analysis

**DOI:** 10.1001/jamaophthalmol.2021.0527

**Published:** 2021-05-13

**Authors:** Abraham D. Flaxman, John S. Wittenborn, Toshana Robalik, Rohit Gulia, Robert B. Gerzoff, Elizabeth A. Lundeen, Jinan Saaddine, David B. Rein

**Affiliations:** 1 Institute for Health Metrics and Evaluation, University of Washington, Seattle; 2 NORC at the University of Chicago, Chicago, Illinois; 3 Applied Statistical Consulting LLC, Atlanta, Georgia; 4 Division of Diabetes Translation, Vision Health Initiative Centers for Disease Control and Prevention, Atlanta, Georgia

## Abstract

**Importance:**

Globally, more than 250 million people live with visual acuity loss or blindness, and people in the US fear losing vision more than memory, hearing, or speech. But it appears there are no recent empirical estimates of visual acuity loss or blindness for the US.

**Objective:**

To produce estimates of visual acuity loss and blindness by age, sex, race/ethnicity, and US state.

**Data Sources:**

Data from the American Community Survey (2017), National Health and Nutrition Examination Survey (1999-2008), and National Survey of Children’s Health (2017), as well as population-based studies (2000-2013), were included.

**Study Selection:**

All relevant data from the US Centers for Disease Control and Prevention’s Vision and Eye Health Surveillance System were included.

**Data Extraction and Synthesis:**

The prevalence of visual acuity loss or blindness was estimated, stratified when possible by factors including US state, age group, sex, race/ethnicity, and community-dwelling or group-quarters status. Data analysis occurred from March 2018 to March 2020.

**Main Outcomes or Measures:**

The prevalence of visual acuity loss (defined as a best-corrected visual acuity greater than or equal to 0.3 logMAR) and blindness (defined as a logMAR of 1.0 or greater) in the better-seeing eye.

**Results:**

For 2017, this meta-analysis generated an estimated US prevalence of 7.08 (95% uncertainty interval, 6.32-7.89) million people living with visual acuity loss, of whom 1.08 (95% uncertainty interval, 0.82-1.30) million people were living with blindness. Of this, 1.62 (95% uncertainty interval, 1.32-1.92) million persons with visual acuity loss are younger than 40 years, and 141 000 (95% uncertainty interval, 95 000-187 000) persons with blindness are younger than 40 years.

**Conclusions and Relevance:**

This analysis of all available data with modern methods produced estimates substantially higher than those previously published.

## Key Points

**Question:**

How many people in the US are living with uncorrectable visual acuity loss or blindness?

**Findings:**

This bayesian meta-analysis generated an estimate that, in 2017, there were 7.08 million people living with visual acuity loss, of whom 1.08 million were living with blindness.

**Meaning:**

Per this study, uncorrectable visual acuity loss and blindness are even larger drivers of health burden in the US than was previously known.

## Teaser

This meta-analysis combines population-based surveys and data from the US Centers for Disease Control and Prevention’s Vision and Eye Health Surveillance System to produce estimates of visual acuity loss and blindness by age, sex, race/ethnicity, and US state.

## Introduction

Globally, an estimated 252.6 (95% CI, 111.4-424.5) million people live with best-corrected visual acuity of 20/60 or worse in the better-seeing eye.^1^ People in the US fear losing vision more than memory, hearing, or speech, and consider visual acuity loss among the top 4 worst things that could happen to them.^2^ No existing estimates appear to have used empirical data to estimate geographic differences, created estimates for persons younger than age 40 years, or accounted for increased prevalence in group quarters.

Previous studies have estimated national visual acuity loss or blindness prevalence for important age ranges. The Vision Problems in the United States (VPUS) study estimated uncorrectable visual impairment and blindness for persons ages 40 years and older to occur in 4.2 million individuals (2.9%) in 2010.^3^ Using similar methods and data for 2015, Varma et al^4^ estimated national and state visual acuity loss or blindness prevalence for persons ages 40 years and older and arrived at a similar estimate of 4.24 million cases (2.8%). Both of these studies^3,4^ are limited, since they excluded persons younger than 40 years and persons living in group quarters, such as nursing homes and prisons. Both studies^3,4^ relied on meta-analytic summaries of similar selected population-based study data, and no other data sources, to estimate prevalence by age group, sex, and race/ethnicity and then calculated state-level estimates by applying these summary estimates to each state’s population distribution. This method may lead to inaccuracies because the population-based study data (while of high quality) were collected 8 to 36 years in the past from locally representative samples using different methods across studies. State-specific estimates assumed that the prevalence of visual acuity loss or blindness observed in the population-based studies were invariant across states, with differences between states resulting only from differences in the included population demographics. However, visual acuity loss or blindness prevalence may vary substantially across states because risk factors for visual impairment (such as diabetes, smoking, sun exposure, nutrition, toxins, or injuries), health care access (eg, health insurance, access to eye care), social determinants of health (eg, poverty, occupational hazards), and policies (eg, school entry screening) vary widely across states.^5,6,7,8^

The Centers for Disease Control and Prevention’s Vision and Eye Health Surveillance System (VEHSS) provides information on diagnosed national and state-specific visual acuity loss or blindness prevalence based on Medicare 100% fee-for-service data, MarketScan private insurance claims, electronic health records from the IRIS Registry (a comprehensive ophthalmology eye diseases clinical registry), and self-reported response data regarding visual difficulty or blindness from 4 national surveys (the America Community Survey [ACS], National Survey of Children’s Health [NSCH], Behavioral Risk Factor Surveillance System, and National Health Interview Survey) and self-reported and examination-based data from the National Health and Nutrition Examination Survey (NHANES). These sources yield divergent prevalence estimates based on differences in case definitions and persons included in the data.^9^ While the VEHSS provides estimates from each of these sources, to our knowledge, no attempt has been made to summarize these data into a single meta-analytic national estimate.

We selected all relevant VEHSS data to create new national and state estimates of US blindness and visual acuity loss for all ages for the year 2017. We used bayesian meta-regression to combine all relevant information from the ACS (for state-to-state variation, the oldest age groups, and prevalence in group quarters), NHANES (a primary source of information for mean tendency, age stratification, sex, and race/ethnicity variation), the NSCH (for individuals of the youngest ages), and population-based studies (PBS), and summarized results by point estimates and uncertainty intervals (UIs).

## Methods

### Ethical Review

These research activities were deemed to be not human subjects research by the institutional review board of NORC at the University of Chicago because they are based exclusively on secondary analysis of existing, deidentified data sources. For this reason, informed consent was not required.

### Strategy

We applied bayesian meta-regression methods^10^ to multiple data sources with the goal of producing estimates of the prevalence and uncertainty interval of visual acuity loss or blindness, stratified by age group, sex, race/ethnicity, and state (50 US states and Washington, DC) for the year 2017. We defined visual acuity loss using US standards as a best-corrected visual acuity greater than or equal to 0.3 logMAR (a Snellen score of 20/40 or worse) and blindness as a subset of that group, consisting of those with a logMAR of 1.0 or greater (a Snellen score of 20/200 or worse) in the better-seeing eye. We first estimated all visual acuity loss of logMAR 0.3 or greater, and then in a second, separate calculation, estimated blindness (logMAR ≥1.0).

### Data

Our model used 4 data sources: (1) data abstracted from PBS, (2) National Health and Nutrition Examination Survey (NHANES) data collected during 1999 to 2008 (the only years in which vision data were collected), (3) ACS data collected in 2017, and (4) NSCH data collected in 2016. For PBS, we searched online sources for studies published after 1991 that were representative of the target population from which the participants were sampled, presented primary results or meta-analysis of primary data, and reported age-specific, race/ethnicity–specific, and/or location-specific prevalence estimates.^11^ We identified 5 such studies for inclusion from (1) the Baltimore Pediatric Eye Disease Study (data collection period, 2003-2007; publication date, 2008)^12^; (2) the Chinese American Eye Study (data collection period, 2010-2013; publication date, 2016)^13^; (3) the Eye Diseases Prevalence Research Group (EDPRG; a meta-analysis of several earlier PBS; data collection period, 1985-1998; publication date, 2004)^14^; (4) the Los Angeles Latino Eye Study data collection period, 2000-2003; publication date, 2004)^15^; and (5) the Multi-Ethnic Study of Atherosclerosis Cohort (data collection period, 2000-2004; publication date, 2015).^16^ We abstracted estimated prevalence of dichotomous measures of visual impairment and blindness and sample size information from each study by age group, sex, and race/ethnicity. Of these sources, all but the EDPRG reported primary data on best-corrected visual acuity, as measured by study ophthalmologists.

For NHANES participants aged 12 years or older, we used eye examination–derived measurements of best-corrected visual acuity, as measured among persons with presenting visual acuity of 20/40 or worse using the Auto Refractor model ARK-760 (Nidek) instrument and collected as part of a visual health module that was fielded from 1999 to 2008 from a nationally representative sample of US individuals dwelling in communities.^17^ Among those with measurements, best-corrected visual acuity was missing for 11.51%. As described in the eMethods in Supplement 1, we imputed missing categorical indicators of visual acuity loss and blindness using multiple imputation with chained equations with bootstrapped resampling.^18^

The NSCH is a nationally representative survey of the physical and emotional health of children aged 0 to 17 years that contains a caregiver-reported assessment of visual difficulty, which reads, “Does this child have blindness or problems with seeing, even when wearing glasses?”^19^ The ACS is an annual nationally representative and state-representative survey conducted by the US Census Bureau to provide information on demographic, social, economic, and housing characteristics of the US population.^20^ Like NSCH, ACS includes a head-of-household–reported assessment of visual difficulty, which reads, “Is this person blind or does he/she have serious difficulty seeing even when wearing glasses?” and for which the respondent reports for all members of the household. The ACS also includes information on group-quartered residences, allowing questions to be analyzed for those in nursing homes, prisons, and other institutional group quarters, separately from residents in community-dwelling households.

### Estimation

We developed 2 statistical models to assess (1) the prevalence rate of all visual acuity loss stratified by age group, sex, race/ethnicity, group-quarters status, and US state and (2) the prevalence rate of blindness at the same levels of stratification. The model estimated the dependent variable, observed prevalence in each stratification category, as a negative, binomially distributed function of the number of persons evaluated in the sample and independent variables measuring sex, age, race/ethnicity (non-Hispanic Black, non-Hispanic White, Hispanic, and other), US state, and source of data. We applied the integrative systems modeling approach developed in the Global Burden of Disease Study to create these estimates.^10^ Following King,^21^ our integrative systems modeling reduces to an extension of negative binomial regression, with a piecewise linear spline to represent the nonlinear age pattern and an age-standardizing likelihood to account for the heterogeneous reporting of age groups in examination study data. This allowed us to include data from all 5 PBS as well as the NHANES, NSCH, and ACS in the likelihood during parameter estimation. We used the DisMod-MR 1.1.1, which implements this model in Python version 3.6 using PyMC 2, and fit the model with 400 000 iterations of Markov chain Monte Carlo using an adaptive metropolis step method.^22^ The model includes parameters to estimate variation in prevalence as a function of age, sex, race/ethnicity, and data source and assumes that the age-stratified prevalence rate is not changing substantially over time. Full details are provided in the eMethods in Supplement 1. Data analysis occurred from March 2018 to March 2020.

## Results

Our data abstracted from PBS consisted of 103 measurements of visual acuity loss and 43 measurements of blindness. The surveys used included 35 466 individuals from the NHANES, 3 190 040 individuals from the ACS, and 50 212 individuals from the NSCH.

### Visual Acuity Loss or Blindness

**Figure 1.**
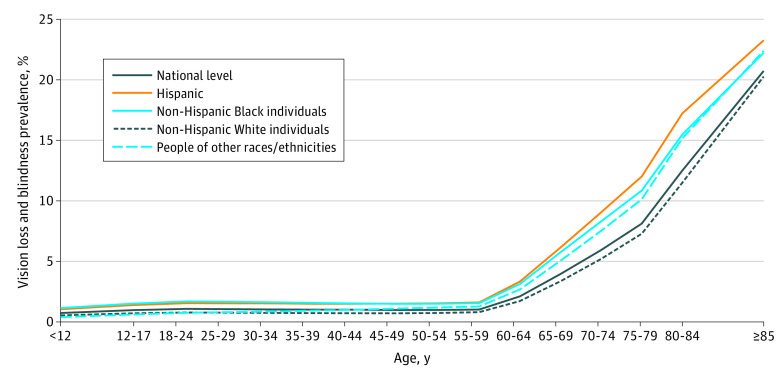
Crude Prevalence of Visual Acuity Loss or Blindness by Age for All Racial/Ethnic Groups Increases appear starting at approximately age 60 years.

We estimated a US prevalence count of 7.08 (95% UI, 6.32-7.89) million people living with visual acuity loss or blindness (using the US standard of best-corrected visual acuity in the worse-seeing eye of a Snellen score of 20/40 or worse) in the US in 2017, corresponding to a crude prevalence rate of 2.17% (95% UI, 1.94%-2.42%) (Table 1). The national prevalence level of visual acuity loss or blindness increased as a function of age, from 0.74% (95% UI, 0.37%-1.10%) among persons younger than 12 years to 0.99% (95% UI, 0.80%-1.18%) among individuals aged 50 to 54 years and 20.73% (95% UI, 17.71%-23.27%) among persons aged 85 years and older (Figure 1).

**Table 1.** Estimated Crude Prevalence Count and Rate of People Living With Visual Acuity Loss or Blindness, Stratified by Sex and Race/Ethnicity, US, 2017

Characteristic	Prevalence count, millions of people			Prevalence rate, %		
	Mean	2.5th Percentile	97.5th Percentile	Mean	2.5th Percentile	97.5th Percentile
Total	7.08	6.32	7.89	2.17	1.94	2.42
Female	4.16	3.62	4.69	2.52	2.19	2.84
Male	2.92	2.53	3.37	1.82	1.57	2.10
Non-Hispanic						
Black	1.02	0.87	1.18	2.55	2.17	2.94
White	4.27	3.68	4.87	2.16	1.86	2.47
Hispanic	1.26	1.07	1.47	2.15	1.83	2.50
Other	0.52	0.41	0.62	1.76	1.40	2.12

Our meta-regression also estimated that 358 000 (95% UI, 263 000-472 000) persons with visual acuity loss or blindness reside in group quarters, such as nursing homes and prisons. This constitutes 5.06% (95% UI, 3.78%-6.60%) of all persons with visual acuity loss or blindness.

We estimated 1.62 (95% UI, 1.32-1.92) million persons with visual acuity loss or blindness are younger than 40 years. This constitutes 22.89% of all persons with visual acuity loss or blindness.

**Figure 2.**
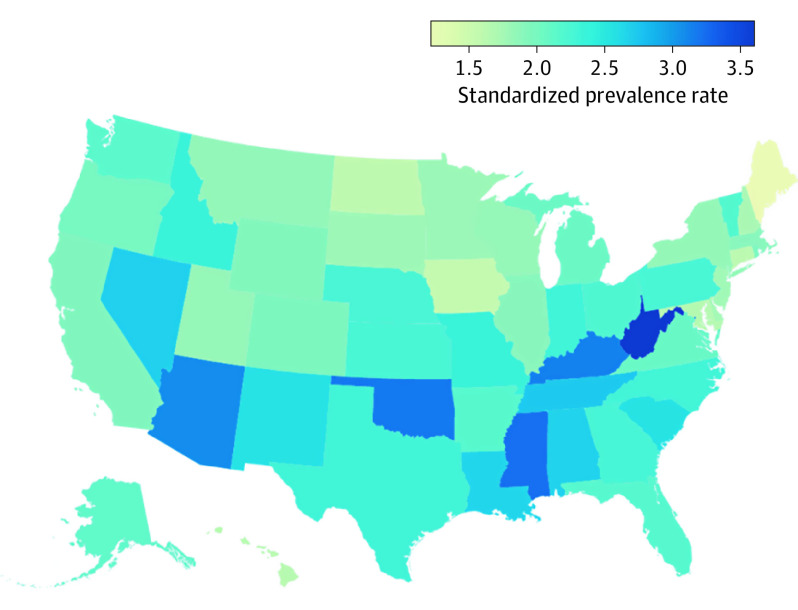
Age-Standardized, Sex-Standardized, and Race/Ethnicity–Standardized Visual Acuity Loss or Blindness Prevalence by State

Crude prevalence rates of visual acuity loss or blindness ranged from 1.35% (95% UI, 1.02%-1.65%) in Maine to 3.59% (95% UI, 2.93%-4.26%) in West Virginia. State differences persisted after standardization by age, sex, and race/ethnicity (Figure 2). Estimated counts and prevalence rates for each US state are provided in eTable 1 in Supplement 1.

### Blindness

As a subset of persons with visual acuity loss or blindness, we estimated a 2017 US prevalence count of 1.08 (95% UI, 0.82-1.30) million people living with blindness, defined as a best-corrected visual acuity of 1.0 logMAR or greater (corresponding to a Snellen score of 20/200 or greater) in the better-seeing eye. This is equal to a crude prevalence rate of 0.33% (95% UI, 0.25%-0.40%) (Table 2). The crude prevalence rate of blindness increased substantially as a function of age, from 0.05% (95% UI, 0.02%-0.08%) among persons 12 years and younger to 0.11% (95% UI, 0.08%-0.15%) among individuals aged 50 to 54 years and 5.50% (95% UI, 3.70%-7.30%) among persons 85 years and older (eFigure in Supplement 1).

**Table 2.** Estimated Prevalence Count of People Living With Blindness, Stratified by Sex and Race/Ethnicity, as Well as Prevalence Rates

Characteristic	Prevalence count, millions of people			Prevalence rate, %		
	Mean	2.5th Percentile	97.5th Percentile	Mean	2.5th Percentile	97.5th Percentile
Total	1.08	0.82	1.30	0.33	0.25	0.40
Female	0.64	0.48	0.79	0.38	0.29	0.48
Male	0.45	0.34	0.55	0.28	0.21	0.35
Non-Hispanic						
Black	0.17	0.13	0.21	0.42	0.32	0.53
White	0.74	0.56	0.92	0.37	0.28	0.47
Hispanic	0.12	0.09	0.16	0.21	0.16	0.27
Other	0.05	0.04	0.08	0.19	0.13	0.26

**Figure 3.**
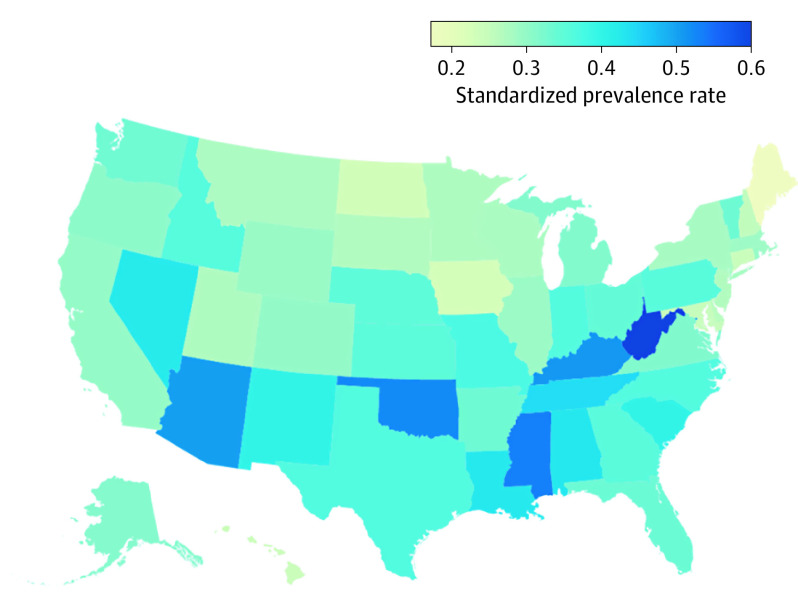
Age-Standardized, Sex-Standardized, and Race/Ethnicity–Standardized Blindness Prevalence Estimates by State

We estimated that 130 000 (95% UI, 57 000-223 000) people with blindness are living in group quarters, such as nursing homes and prisons. This constitutes 11.85% (95% UI, 5.52%-18.76%) of all people living with blindness. We estimated 141 000 (95% UI, 95 000-187 000) persons with blindness are younger than 40 years, which constitutes 13.09% of all persons with blindness. Crude prevalence rates of blindness ranged from 0.19% (95% UI, 0.14%-0.25%) in Utah to 0.65% (95% UI, 0.46%-0.83%) in West Virginia (eTable 2 in Supplement 1; Figure 3).

## Discussion

Our estimated number of cases of visual acuity loss or blindness is 68.7% higher than the previous estimate created by the VPUS study, but our estimate of blindness alone is lower. Although the VPUS study reported findings among people 40 years and older based on different source data, this increase in estimated visual acuity loss or blindness is largely the result of our inclusion of the NHANES data in the model and our choice to use imputation instead of listwise deletion to address the missing NHANES data. We estimated higher prevalence for Hispanic and Black individuals compared with White individuals and for women compared with men; however, at least some of these estimates are very uncertain, with a posterior probability distribution that crosses zero. These results are consistent with previous analyses of NHANES data, which also found a higher risk of visual acuity loss among Hispanic and Black individuals compared with White individuals and in women compared with men but were not able to conclude that these higher risks were statistically significant.^23^ Other important differences between our estimate and the VPUS estimate include (1) using more recent population-based study data; (2) using 2017 population structure for age, sex, race/ethnicity, and household or group quarters size; (3) accounting for differences in prevalence in populations in community-dwelling households vs group quarters; and (4) accounting for variations across states.

### Limitations

Our analyses were limited by at least 5 factors. First, the NHANES data had a substantial amount (approximately 12%) of missing autorefractor examination data. Our method of accounting for missing data, multiple imputations by chained equation, resulted in a substantially higher estimate of the prevalence rate of visual acuity loss (2.1%) than is obtained using the same data and listwise deletion (1.7%). While we believe multiple imputations by chained equation is the superior method to handle missing data because it uses the strength of other information to inform the estimates, less missing data in NHANES would have resulted in more precise estimates.

Second, our estimates may be limited by the age of some of the included data sets. The NHANES data were collected from 1999 to 2008, and data from some of the population-based examination studies included in the EDPRG meta-analyses were collected even prior to that. However, our model also included more recent PBS published after the EDPRG meta-analysis, as well as the 2016 NSCH and the 2017 ACS. Additionally, time-trend analyses of the ACS did not indicate systematic differences in age-stratified, sex-stratified, or race/ethnicity–stratified vision prevalence between the years 2008 and 2017 (not shown).

Third, we used survey respondent–reported values from the ACS to account for differences in visual acuity loss prevalence at the state level and within group quarters and from the NSCH for children. Since these values are not based on an examination, they likely contain false-positive results at least for uncorrected refractive error. Our model corrects for systematically higher prevalence in self-reported visual difficulty measures. However, to estimate prevalence variation by state, household status, and childhood ages, our model assumes that examination data on best-corrected visual acuity, if it were collected, would vary following the same pattern as these self-reported data. Furthermore, because the ACS data included only a single measure of severe visual difficulty or blindness, our model assumes that state variation is the same for both visual acuity loss and blindness together as for blindness alone and by household status. We believe this assumption is both reasonable and currently necessary to create data-driven estimates for state, residents in group quarters, and children, but we acknowledge that additional examination data within these strata would improve the quality of future estimates.

Fourth, we have assumed that the decomposition of visual acuity loss in distinct subcategories of visual impairment and blindness follow the same percentage breakdown in group quarters as in households. Although it seems plausible that the fraction of visual acuity loss that is blindness is higher in group quarters than in the household population, we found no reliable, representative data source to test this hypothesis or quantify the magnitude of the difference. The Medicare Minimum Data Set is generated as part of a clinical assessment of all residents in Medicare-certified or Medicaid-certified nursing home, and includes an assessment of each resident’s functional capabilities and health needs. However, it does not collect data on visual difficulty in a format that we were able to integrate into our model. The Baltimore Nursing Home Eye Survey^24^ found that 47% of people living with visual acuity loss in nursing homes were blind (compared with our finding of 14.5%), and if we generalize this 47% to the entire group-quarters population, we expect an additional 118 000 (95% UI, 87 000-156 000) people living with blindness.

Finally, we estimated visual acuity loss or blindness independently, despite the logical interdependency that every person living with blindness is, by definition, a person living with visual acuity loss. A more complex model that estimated the 2 outcomes simultaneously could perhaps make more efficient use of the sparse data available and eliminate the illogical possibility of estimating more people living with blindness than living with visual acuity loss. However, the model structure presented here resulted in no instances in which the estimated rate of blindness exceeded the estimated rate of visual acuity loss at any level of stratification.

There are also several data sources in the VEHSS that we were not able to include in our analysis. Medicare and MarketScan claims data and IRIS registry data are both appealing big data sources, but we were not able to adjust for the nonrepresentative nature of the populations represented by these sources, and there is a lack of evidence on the validity of diagnosed vision loss as a measure of population-level vision. The Behavioral Risk Factor Surveillance System and National Health Interview Survey both include respondent-reported data similar to ACS, which we excluded on our assessment that the ACS sample size is substantially larger and the potential biases of respondent-reported visual acuity loss would be compounded by bringing together sources from multiple instruments and surveys.

## Conclusions

Visual acuity loss and blindness continue to be a substantial burden to the US population, and our new analysis indicates that the issue is even more substantial than has previously been recognized. Efforts to collect new examination-based information on best-corrected visual acuity in the better-seeing eye would enhance future efforts to create more precise national and state estimates of visual acuity loss or blindness, and this evidence base could be valuable for targeted efforts to prevent or treat these conditions.

## Notes

Supplement 1. **eMethods.** Details, verification, and validation of our methodological approach. **eFigure.** Crude prevalence rate of blindness increases with age for all race and ethnic groups, starting around age 60 years. **eTable 1.** Estimated prevalence count of people living with visual acuity loss or blindness, stratified by state, as well as prevalence rates (in percent). **eTable 2.** Estimated prevalence count of people living with blindness, stratified by location, as well as prevalence rates (in percent).

Supplement 2. **Nonauthor Collaborators.** Vision and Eye Health Surveillance System Study Group members.
