# Supplementary material for: Prevalence of Visual Acuity Loss or Blindness in the US: A Bayesian Meta-analysis
Source: JAMA Ophthalmol. 2021 May 13;139(7):717–23. doi: 10.1001/jamaophthalmol.2021.0527 (PMC8120442; doi:10.1001/jamaophthalmol.2021.0527)
Supplement: Supplement 1. — eMethods. Details, verification, and validation of our methodological approach. eFigure. Crude prevalence rate of blindness increases with age for all race and ethnic groups, starting around age 60 years. eTable 1. Estimated prevalence count of people living with visual acuity loss or blindness, stratified by state, as well as prevalence rates (in percent). eTable 2. Estimated prevalence count of people living with blindness, stratified by location, as well as prevalence rates (in percent). [file jamaophthalmol-e210527-s001.pdf]

Supplemental Online Content

Flaxman AD, Wittenborn JS, Robalik T, et al; the Vision and Eye Health Surveillance System Study Group. Prevalence of visual acuity loss or blindness in the US: a bayesian meta-analysis. *JAMA Ophthalmol*. Published May 13, 2021. doi:10.1001/jamaophthalmol.2021.0527

eMethods. Details, verification, and validation of our methodological approach.

eFigure. Crude prevalence rate of blindness increases with age for all race and ethnic groups, starting around age 60 years.

eTable 1. Estimated prevalence count of people living with visual acuity loss or blindness, stratified by state, as well as prevalence rates (in percent).

eTable 2. Estimated prevalence count of people living with blindness, stratified by location, as well as prevalence rates (in percent).

This supplemental material has been provided by the authors to give readers additional information about their work.

© 2021 Flaxman AD et al. *JAMA Ophthalmology.*

eMethods: details, verification, and validation of methodological approach

In this supplementary appendix, we present additional details on the verification and validation of our methodological approach. To better understand the impact of modeling choices, we examined a nested sequence of intermediate models of increasing complexity.

Initially we estimated the numbers of cases of visual acuity loss using only PBS data. We then added NHANES data as reference with PBS data for the estimation, and then merged NSCH data with NHANES data and PBS data in the model and generated estimates of visual acuity loss. We then added ACS data for older ages and group quarters sequentially (denoted ACS-older and ACS-gq in Table 1 below). After that we used ACS data for state-specific prevalence and for year 2017 to generate estimates (denoted ACS-state and ACS-2017 in Table 1 below). In the final model we added interaction terms along with all the datasets to generate visual acuity loss estimates.

Table 1: Estimated prevalence counts (in millions) of people living with visual acuity loss or blindness, after each step of the sequence in the model.

Data Sources	Visual acuity loss among age 40+ (in millions)	Visual acuity loss in all ages (in millions)
Population Based Study data (Exam)	2.92 [2.12, 3.83]	3.47 [2.56, 4.61]

© 2021 Flaxman AD et al. *JAMA Ophthalmology.*

Exam, NHANES	4.28 [3.41, 5.25]	5.80 [4.78, 6.97]
Exam, NHANES, NSCH	4.35 [3.50, 5.30]	5.83 [4.81, 6.92]
Exam, NHANES, NSCH, ACS-older	4.49 [3.63, 5.47]	5.98 [5.07, 7.08]
Exam, NHANES, NSCH, ACS-older, ACS-gc	4.63 [3.85, 5.48]	6.16 [5.29, 7.13]
Exam, NHANES, NSCH, ACS-older, ACS-gc, ACS-state	4.57 [4.02, 5.16]	6.11 [5.38, 6.89]
Exam, NHANES, NSCH, ACS-older, ACS-gc, ACS-state, ACS-2017	5.43 [4.77, 6.16]	7.04 [6.29, 7.92]
Exam, NHANES, NSCH, ACS-older, ACS-gc, ACS-state, ACS-2017, interaction terms	5.46 [4.83, 6.16]	7.08 [6.34, 7.92]

Table 2 presents similar results for the subset of patients who we predicted to be blind.

Table 2: Estimated prevalence counts (in millions) of people living with blindness, after each step of the sequence in the model.

© 2021 Flaxman AD et al. *JAMA Ophthalmology.*

Data Sources	Blindness among age 40+ (in millions)	Blindness in all ages (in millions)
Population Based Study data (Exam)	0.73 [0.48, 1.10]	0.77 [0.51, 1.15]
Exam, NHANES	0.71 [0.45, 1.14]	0.82 [0.56, 1.25]
Exam, NHANES, NSCH	0.73 [0.48, 1.07]	0.86 [0.59, 1.20]
Exam, NHANES, NSCH, ACS-older	0.69 [0.50, 0.94]	0.82 [0.60, 1.08]
Exam, NHANES, NSCH, ACS-older, ACS-gc	0.81 [0.59, 1.09]	0.96 [0.71, 1.24]
Exam, NHANES, NSCH, ACS-older, ACS-gc, ACS-state	0.81 [0.62, 1.04]	0.96 [0.74, 1.21]
Exam, NHANES, NSCH, ACS-older, ACS-gc, ACS-state, ACS-2017	0.91 [0.71, 1.12]	1.06 [0.84, 1.28]
Exam, NHANES, NSCH, ACS-older, ACS-gc, ACS-state, ACS-2017, interaction terms	0.93 [0.74, 1.17]	1.08 [0.85, 1.34]

© 2021 Flaxman AD et al. *JAMA Ophthalmology.*

Readers may notice that our reported results when using only PBS data (row 1 of Tables 1 and 2) are lower than those reported by the Vision Problems in the U.S. (VPUS) study which was also based on population-based studies (3). That study (available at visionproblemsus.org) estimated 4.19 million people with vision impairment and blindness, of whom 1.29 million were blind. Based only on population-based studies, our model estimates 2.92 million people who had visual acuity loss or blindness, of whom approximately 730,000 were blind. This difference is driven by the PBS that were used in our model as compared to the earlier VPUS. The studies we included as PBS are described in the data section above. They can be compared to those used by VPUS by reviewing the VPUS Methods and Sources page. In general, the studies that we included reported a lower prevalence of visual impairment and blindness than the studies included in the VPUS and this lower prevalence is reflected in our model estimate.

We believe specific differences between our estimates are attributable to the inclusion of older data from the Baltimore Eye Survey (collected from 1985 and 1988) *(1)* and the Salisbury Eye Evaluation Project (collected from 1993 and 1995) *(2)* in VPUS, and our inclusion of data from CHES (collected from 2010-2013), LALES (collected from 2000-2008), and MESA (collected from 2002-2004).

MICE-Multiple imputation by chained equation

Missing data in meta-analysis can jeopardize inferences from the study and examination data, and possibly bias the estimates. The vision examination data from NHANES is a key input in our meta-analytic estimates of the prevalence of uncorrectable visual acuity loss or blindness in the United States, but when we mapped this data to dichotomous outcomes for visual acuity loss or blindness, we were unable to determine a value for 11.51% of respondents.

© 2021 Flaxman AD et al. *JAMA Ophthalmology.*

Multiple imputation by chained equations (MICE) is a standard approach to address missing data. MICE constructs a regression model for each column with missing values, using the variables from the other columns as the predictors. If multiple columns have missing values, the MICE procedure iterates through the columns, fitting each with the previously imputed values for the other columns. Simpler approaches, such as complete-case analysis (also called list-wise deletion) and available-case analysis, may produce biased results. The mean age among individuals with incomplete eye exam was 52.8 years, substantially higher than the mean age among individuals with complete eye exam (38.6 years), and since visual acuity loss or blindness prevalence rates increase markedly as age increases, our imputation method must take age into account.

Using equation 1 and 2 below, we imputed missing dichotomous indicators of visual loss and blindness using multiple imputation with chained equations (MICE) with Gaussian perturbations:

$$\text{Visual acuity loss} \sim \mathrm{C(age\_group)} + \mathrm{sex} + \mathrm{C(race\_eth)} + \mathrm{vidrva} + \mathrm{vidlva}, \quad (1)$$

$$\text{Blindness} \sim \mathrm{C(age\_group)} + \mathrm{sex} + \mathrm{C(race\_eth)} + \mathrm{vidrva} + \mathrm{vidlva}, \quad (2)$$

where vidrva and vidlva are presenting visual acuity of the right eye and left eye respectively, C(age_group) respresents a "dummy coding" for age variable grouped in bins (0, 12, 18, 25, 30, 35, 40, 45, 50, 55, 60, 65, 70, 75, 80, 85, 100) and C(race_eth) represents a "dummy coding" for our categorical variable for race/ethnicity.

The MICE approach requires some choices: what else should go into the regression formula for the missing variables? Should we use a Gaussian for perturbing the model parameters in the imputation model, or a non-parametric bootstrap? To identify the best

© 2021 Flaxman AD et al. *JAMA Ophthalmology.*

regression formula and perturbation method for imputing missing values of visual acuity loss or blindness, we conducted an out-of-sample validation exercise.

We considered a range of regression equations for our outcomes of interest (visual acuity loss or blindness): (in the formulas below, the notation (var1 + var2)**2 means the variables var1 and var2 are included as main effects and all pair-wise interactions are also included)

1. outcome ~ age + sex + C(race) (where C(race) is a "dummy" coding of the race/ethnicity variable)
2. outcome ~ C(age_group) + sex + C(race) (where C(age_group) is a "dummy" coding of the age variable grouped in bins [0, 12, 18, 25, 30, 35, 40, 45, 50, 55, 60, 65, 70, 75, 80, 85, 100])
3. outcome ~ C(age_group) + sex + C(race) + vidrva (where vidrva is the presenting visual acuity in the right eye)
4. outcome ~ C(age_group) + sex + C(race) + vidlva (where vidlva is the presenting visual acuity in the left eye)
5. outcome ~ C(age_group) + sex + C(race) + vidrva + vidlva
6. outcome ~ C(age_group) + sex + C(race) + (vidrva + vidlva)**2
7. outcome ~ C(age_group) + sex + C(race) + vidrova (where vidrova is the best-corrected visual acuity in the right eye)
8. outcome ~ C(age_group) + sex + C(race) + vidlova (where vidlova is the best-corrected visual acuity in the left eye)
9. outcome ~ C(age_group) + sex + C(race) + vidrova + vidlova
10. outcome ~ C(age_group) + sex + C(race) + (vidrova + vidlova)**2

© 2021 Flaxman AD et al. *JAMA Ophthalmology.*

11. outcome ~ C(age_group) + sex + C(race) + vidrva + vidlva + vidrova + vidlova
12. outcome ~ C(age_group) + sex + C(race) + (vidrva + vidlva + vidrova + vidlova)**2

We tested each equation for each outcome for two alternative perturbation methods: (a) the Gaussian perturbation method, which samples from a multivariate normal distribution derived from the fit of the regression equation; and (b) the bootstrap perturbation method, which samples from the conditional model fitted to a bootstrapped version of the data set.

For our testing approach, we used an out-of-sample cross-validation approach from machine learning, where we withheld the presenting and best corrected visual acuity measurements for a randomly selected subset of data (our "test dataset") and compared the model predictions for these individuals to the true values of blindness and visual acuity loss.(3) To be precise,

1. First, we selected 25% of the rows of NHANES data and redacted their vidrva, vidlva, vidrova, and vidlova values (which are all numeric values measured for presenting and best corrected visual acuity in the right and left eye; these values are always sufficient to derive the values of the visual acuity loss and blindness variables, and certain patterns of missing values among these measurements lead to missing values of the dichotomous visual acuity loss and blindness variables). For these rows, we also redacted the visual acuity loss or blindness values derived from these measurements.
2. Second, we imputed the masked values using MICE 1,000 times and took the average of these values as a probability prediction of the outcome of interest (visual acuity loss or blindness).

© 2021 Flaxman AD et al. *JAMA Ophthalmology.*

3. Third, we compared those imputed values and actual values using the ROC curve in which the true positive rate (Sensitivity) is plotted as a function of the false positive rate (100-Specificity) for different cut-off points of a parameter. Each point on the ROC curve represents a sensitivity/specificity pair corresponding to a particular decision threshold. We measured the area under the ROC curve (AUC) as a summary metric for performance of the MICE formulation.

MICE validation results

Missing values were best imputed using the variables age, sex, race, vidrva and vidlva, as shown in Table 3.

Table 3: Table showing the AUC score of all the models with different parameters

Method / Equation	VL Gaussian	VL Bootstrap	Blindness Gaussian	Blindness Bootstrap
Age + Sex + C(Race)	70.11	69.74	73.15	72.95
C(Age_group) + Sex + C(Race)	72.21	71.18	72.51	70.39
C(Age_group) + Sex + C(Race) + Vidrva	75.81	75.86	80.14	81.09
C(Age_group) + Sex + C(Race) + Vidlva	74.72	75.66	77.05	83.89
C(Age_group) + Sex + C(Race) + Vidrva + Vidlva	**75.51**	74.66	**80.75**	78.53

© 2021 Flaxman AD et al. *JAMA Ophthalmology.*

C(Age_group) + Sex + C(Race) + (Vidrva + Vidlva)**2	74.98	75.74	78.70	78.93
C(Age_group) + Sex + C(Race) + Vidrova	75.04	74.49	82.95	70.89
C(Age_group) + Sex + C(Race) + Vidlova	75.29	74.81	77.66	79.59
C(Age_group) + Sex + C(Race) + Vidrova +Vidlova	74.19	74.99	75.22	72.04
C(Age_group) + Sex + C(Race) + (Vidrova + Vidlova)**@	74.25	72.43	69.24	75.48
C(Age_group) + Sex + C(Race) + Vidrva + Vidlva + Vidrova + Vidlova	74.66	72.97	70.27	67.53
C(Age_group) + Sex + C(Race) + (Vidrva + Vidlva + Vidrova + Vidlova)**2	72.60	71.26	77.00	78.62

Our selected approach to MICE produced out-of-sample AUC of 75.51, compared with AUC of 70.79 in complete-case analysis.

Bayesian meta-regression model

We represent our model with a stochastic component and a systematic component, where the stochastic component is a negative binomial model of count data:

$$(3) \qquad Y_i \sim \text{NegativeBinomial}\left(N_{\text{eff}}^{i}, \pi_i, \eta\right),$$

© 2021 Flaxman AD et al. *JAMA Ophthalmology.*

where i indexes the specific measurement, Y_i is the prevalence count of those with visual acuity loss (or blindness) in measurement i, N_{eff}^i is the effective sample size from which the count was taken (and so $p_i = \frac{Y_i}{N_{\text{eff}}^i}$ is the prevalence rate typically reported in a PBS), π_i is the prevalence rate predicted by the model, η is the over-dispersion parameter of the negative binomial distribution (assumed to be the same for all measurements). DisMod-MR 1.1.1 uses the Python PyMC2 package to implement this Bayesian computation, and follows the formulation of the negative binomial model provided by PyMC2, where $\Pr(Y = x|\mu, \alpha) = \frac{\Gamma(x+\alpha)}{x!\,\Gamma(\alpha)}\left(\frac{\alpha}{\mu+\alpha}\right)^{\alpha}\left(\frac{\mu}{\mu+\alpha}\right)^{x}$, which in terms of the equation above has $x = p_i \cdot N_{\text{eff}}^i, \mu = \pi_i,$ and $\alpha = \eta$.

In the systematic component of the model, we included fixed effects for sex (α_{sex}), age (β_k for $k = 0, \ldots, K$), race (γ_j for $j = \text{white, Black, Hispanic, and other races}$), and data source ($\delta_j$ for j =EDPRG, CHES, LALES, ACS, BPEDS, MESA, and NSCH; we coded NHANES as the reference category), as well as sex/race and race/age interaction fixed effects. We also include random effects for state (50 states and Washington, D.C.; η_j for $j = 1, \ldots, 51$). Our formulation includes a piece-wise linear spline model on age, with spline knots (knot_1,knot_2,…) indexed by k for $k = 1, \ldots, K$, as well as intercept shifts for additional key covariates to account for differential sex ratios and age patterns by race as follows:

(4) $$\pi_i = \exp\{a_{\text{sex}}[\text{sex}_i = \text{female}]\} \times \left(\int_{x=\text{age}_0^i}^{\text{age}_1^i} \left(\beta_0 + \sum_{k=1}^{K} \beta_k \,(x - \text{knot}_k)^+\right) dx\right)$$

$$\times \exp\left\{\sum_{j=1}^{4} \gamma_j \,[\text{race}_i = j] + \sum_{j=1}^{7} \delta_j \,[\text{source}_i = j] + \sum_{j=1}^{51} \eta_j \,[\text{location}_i = j] + \text{interaction\_terms}\right\},$$

where sex_i is the sex measured in measurement i; age_0^i and age_i^i are the start and end of the age group measured in measurement i; race_i is the race/ethnicity group measured in measurement i; source_i is the data source for measurement i; location_i is the state location of measurement i; and notation $[\text{variable} = \text{value}]$ represents an indicator function which takes value 1.0 if the variable is equal to the value and 0.0 otherwise and notation $(\text{value})^+$ represents the value in the parenthesis if it is positive, and takes value zero, otherwise. We used evenly spaced spline knots at ages (0, 20, 40, 60, 80, 100).

We also included interaction terms to capture the possibility that the sex and age effects were different by race/ethnicity and group quarters status:

$$\text{interaction\_terms} = \sum_{j=1}^{4} \gamma_j' \,[\text{race}_i = j \text{ and } \text{sex}_i = \text{female}]$$

$$+\gamma_j''[\text{race}_i = j](\text{age}_{\text{mid}}^i) + \gamma_j'''[\text{race}_i = j \text{ and } \text{age}_{\text{mid}}^i > 50](\text{age}_{\text{mid}}^i - 50)$$

$$+\gamma_j''''[\text{g.quarters}_i = \text{inst}](\text{age}_0^i) + \gamma_j'''''[\text{g.quarters}_i = \text{inst}][\text{age}_0^i > 50](\text{age}_0^i - 50),$$

where $\gamma_j{}'$ is the effect coefficient for race j interacted with sex (for j = 1,…4), $\gamma_j{}''$ is the effect coefficient for race j interacted with age, $\gamma_j{}'''$ is the effect coefficient for race j interacted with age > 50, and $\text{age}_{\text{mid}}^i = \frac{\text{age}_0^i + \text{age}_1^i}{2}$ is the midpoint of the age group measured in measurement i. Together $\gamma_j{}''''$ and $\gamma_j{}'''''$ constitute an age-dependent spline for the group quarters population with knots at 0 and 50. We used a Bayesian framework for inference with weakly informative priors for model parameters other than state to assist in regularization, which primarily allowed the data to inform the model estimates. Prior distributions for α_{sex}, β_k, γ_j, $\gamma_j{}'$, $\gamma_j{}''$, $\gamma_j{}'''$,$\gamma_j{}''''$,$\gamma_j{}'''''$ and δ_j were all set to independent normal distributions with mean 0.0 and

standard deviation 1.0. To capture state variation in prevalence, we used informative priors for state random effects, which were informed by the state-to-state variation in age-/sex-/race-standardized endorsement rates of the respondent-reported visual acuity loss question in ACS. This prior took the form

$$(5) \qquad u_j \sim \mathrm{Normal}\left(\mu_j, \sigma_j^2\right),$$

where, in short, μ_j is the log of the ratio of the standardized state prevalence to the national prevalence, and σ_j^2 is the standard deviation of the log-ratio of the crude state prevalence to the national prevalence for all strata with at least 500 individuals, and u_j is normal distribution with mean μ_j and standard deviation σ_j. To be precise, to estimate μ_j and σ_j^2, we first estimated the crude prevalence rate of respondent-reported visual acuity loss in the US in 2017 as observed in ACS data. We next estimated the prevalence rate of respondent-reported visual acuity loss for each state j from the same data source, stratified by age-group, sex, race/ethnicity, and group quarters status. We then used the state-specific stratified estimates to calculate a standardized prevalence rate, standardizing with the age group, sex, race/ethnicity, and group-quarters weights at the national level, to control for demographic difference between states. We then used these data to estimate the ratio of the standardized state prevalence to the national prevalence. For each state j, we set the prior on u_j to be normally distributed with mean $\mu_j = \log\left(\mathrm{ratio}_j\right)$, where ratio_j is the ratio of standardized prevalence in state j to the national prevalence. We obtained an informative standard deviation σ_j for this prior as follows: analogous to the ratio use used for the mean, we constructed a ratio of the national prevalence rate to the state-specific prevalence rate for each strata (stratified by age-group, sex, race/ethnicity, and group quarters status) and calculated the standard deviation of the log-ratio for all strata with at least 500 individuals in the

© 2021 Flaxman AD et al. *JAMA Ophthalmology.*

state, age, sex, and race/ethnicity-specific strata. We put a cap on the predicted prevalence at 0.25 to ensure that we are not over estimating our results.

Only data from ACS was available to inform the group-quarters effect coefficients, but unlike the state-to-state variation, we used this ACS data in the likelihood instead of constructing an informative prior. To guard against inferring with more confidence than appropriate from the ACS group quarters data, we used aggregate measurements for broad age groups (25-year intervals) and did not stratify them by sex or by race/ethnicity. We also included fixed effects for each age group, which effectively treated each comparison of the prevalence in the institutional group quarters population and the free-living population as a separate study in the meta-regression.

We included all other data sources (NHANES, PBS, NSCH) in the likelihood with the assumption that they applied to a nation-level estimate, which we assumed to be constant over time. This includes data from NSCH, NHANES, and BPEDS for children under the age of 18, and also ACS data on free-living adults over the age of 85.

Validation and Verification

We assessed the cross-model validation of our model by setting its population parameters to those of the VPUS study (persons 40 and older in household settings), estimating the number of cases it generated when using only PBS data, and then sequentially estimating the number of cases it generated using the new data sources and model specifications that we included in our final model. See eMethods supplementary materials for full details.

References

© 2021 Flaxman AD et al. *JAMA Ophthalmology.*

1. Tielsch JM, Sommer A, Witt K, Katz J, Royall RM. Blindness and visual impairment in an American urban population: The Baltimore eye survey. Archives of ophthalmology. American Medical Association; 1990;108:286–90.

2. Munoz B, West SK, Rubin GS, Schein OD, Quigley HA, Bressler SB, et al. Causes of blindness and visual impairment in a population of older americans: The Salisbury eye evaluation study. Archives of ophthalmology. American Medical Association; 2000;118:819–25.

3. Kuhn M, Johnson K. Applied Predictive Modeling. Springer Science & Business Media; 2013. 595 p.

© 2021 Flaxman AD et al. *JAMA Ophthalmology.*

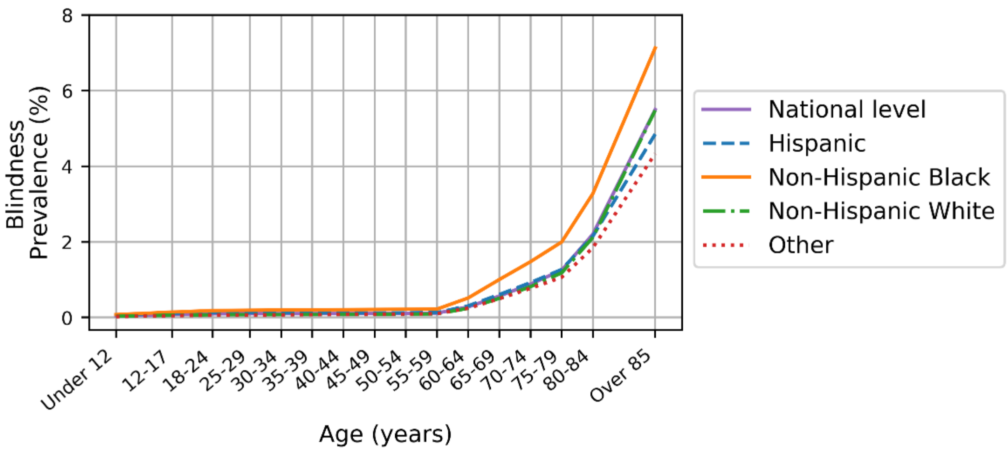

eFigure 1: Crude prevalence rate of blindness increases with age for all race and ethnic groups, starting around age 60 years.

© 2021 Flaxman AD et al. *JAMA Ophthalmology.*

eTable 1: Estimated prevalence count of people living with visual acuity loss or blindness, stratified by state, as well as prevalence rates (in percent).

	Prevalence Count			Prevalence Rate (%)		
	Mean	2.5th percentile	97.5th percentile	Mean	2.5th percentile	97.5th percentile
AK	12,300	10,600	14,400	1.66	1.43	1.95
AL	131,200	112,400	151,100	2.69	2.31	3.10
AR	160,300	135,000	184,300	2.28	1.92	2.63
AZ	89,300	75,800	104,100	2.97	2.52	3.46
CA	810,900	709,900	935,100	2.05	1.80	2.37
CO	99,000	84,200	115,600	1.76	1.50	2.06
CT	62,300	51,500	73,000	1.74	1.44	2.03
DC	22,400	19,400	25,300	3.22	2.80	3.65
DE	18,100	15,400	21,300	1.88	1.60	2.21
FL	558,600	475,700	641,500	2.66	2.27	3.06
GA	221,300	190,900	255,700	2.12	1.83	2.45
HI	30,200	24,500	35,600	2.12	1.71	2.50
IA	48,000	38,800	56,400	1.52	1.24	1.79
ID	37,000	30,600	43,500	2.15	1.78	2.53
IL	244,900	211,600	282,000	1.91	1.65	2.20
IN	141,900	120,400	166,400	2.13	1.81	2.50

© 2021 Flaxman AD et al. *JAMA Ophthalmology.*

KS	63,000	52,600	73,200	2.16	1.80	2.51
KY	125,300	103,600	145,200	2.81	2.33	3.26
LA	123,000	104,600	141,100	2.63	2.23	3.01
MA	129,600	107,200	152,400	1.89	1.56	2.22
MD	102,800	86,900	118,900	1.70	1.44	1.97
ME	18,000	13,600	22,100	1.35	1.02	1.65
MI	200,700	168,100	234,800	2.02	1.69	2.36
MN	92,500	74,900	108,600	1.66	1.34	1.95
MO	138,000	115,500	161,200	2.26	1.89	2.64
MS	98,300	84,100	113,200	3.29	2.82	3.79
MT	19,100	15,500	23,100	1.82	1.47	2.20
NC	230,800	194,000	265,300	2.25	1.89	2.58
ND	12,100	10,000	14,500	1.60	1.32	1.92
NE	40,600	34,400	48,200	2.12	1.79	2.51
NH	23,500	18,900	28,000	1.75	1.41	2.09
NJ	169,000	143,300	192,600	1.88	1.59	2.14
NM	62,300	54,000	71,000	2.98	2.58	3.40
NV	80,300	69,300	91,700	2.68	2.31	3.06
NY	393,200	337,500	449,900	1.98	1.70	2.27
OH	251,200	214,200	296,700	2.15	1.84	2.54

© 2021 Flaxman AD et al. *JAMA Ophthalmology.*

OK	116,400	99,900	135,200	2.96	2.54	3.44
OR	78,900	65,500	93,300	1.90	1.58	2.25
PA	302,600	250,500	352,500	2.36	1.96	2.75
RI	21,500	17,900	25,700	2.03	1.69	2.42
SC	129,800	111,100	149,800	2.58	2.21	2.98
SD	16,000	13,000	18,700	1.84	1.50	2.15
TN	172,300	143,100	199,400	2.57	2.13	2.97
TX	634,000	551,100	726,300	2.24	1.95	2.57
UT	43,000	35,900	50,000	1.39	1.16	1.61
VA	168,100	143,100	191,300	1.98	1.69	2.26
VT	13,900	11,500	16,500	2.23	1.85	2.65
WA	144,200	118,700	165,400	1.95	1.60	2.23
WI	101,600	83,500	119,200	1.75	1.44	2.06
WV	65,200	53,200	77,400	3.59	2.93	4.26
WY	10,200	8,400	12,100	1.77	1.45	2.08

© 2021 Flaxman AD et al. *JAMA Ophthalmology.*

eTable 2: Estimated prevalence count of people living with blindness, stratified by location, as well as prevalence rates (in percent).

	Prevalence Count			Prevalence Rate (%)		
	Mean	2.5th percentile	97.5th percentile	Mean	2.5th percentile	97.5th percentile
AK	1,600	1,200	2,100	0.22	0.17	0.28
AL	21,500	16,400	27,600	0.44	0.34	0.57
AR	22,400	16,700	29,300	0.32	0.24	0.42
AZ	15,300	11,200	19,300	0.51	0.37	0.64
CA	106,200	83,600	131,000	0.27	0.21	0.33
CO	14,000	9,900	17,600	0.25	0.18	0.31
CT	10,200	7,300	13,600	0.28	0.20	0.38
DC	3,700	2,800	4,600	0.53	0.40	0.66
DE	2,900	2,100	3,600	0.30	0.22	0.38
FL	84,800	64,600	108,800	0.40	0.31	0.52
GA	33,300	25,500	41,400	0.32	0.24	0.40
HI	3,800	2,500	5,100	0.27	0.18	0.36
IA	8,100	5,300	10,900	0.26	0.17	0.35
ID	5,600	4,000	7,200	0.33	0.23	0.42
IL	38,200	27,900	48,700	0.30	0.22	0.38
IN	23,400	16,800	30,500	0.35	0.25	0.46

© 2021 Flaxman AD et al. *JAMA Ophthalmology.*

KS	10,600	7,600	14,100	0.37	0.26	0.48
KY	21,300	15,900	27,700	0.48	0.36	0.62
LA	20,100	15,100	24,900	0.43	0.32	0.53
MA	20,600	14,400	27,100	0.30	0.21	0.40
MD	15,700	12,000	20,300	0.26	0.20	0.34
ME	3,000	2,000	4,000	0.22	0.15	0.30
MI	32,300	23,500	41,900	0.32	0.24	0.42
MN	15,400	10,300	20,300	0.28	0.18	0.36
MO	23,200	16,000	30,400	0.38	0.26	0.50
MS	16,700	13,100	20,800	0.56	0.44	0.70
MT	3,000	2,100	4,000	0.29	0.20	0.38
NC	37,000	27,800	46,400	0.36	0.27	0.45
ND	2,100	1,400	2,900	0.28	0.19	0.38
NE	7,000	5,000	9,200	0.36	0.26	0.48
NH	3,900	2,600	5,200	0.29	0.19	0.39
NJ	25,400	19,000	31,900	0.28	0.21	0.35
NM	8,300	6,500	10,600	0.40	0.31	0.51
NV	10,900	8,200	13,400	0.36	0.28	0.45
NY	59,800	45,800	75,900	0.30	0.23	0.38
OH	42,500	31,500	55,600	0.36	0.27	0.48

© 2021 Flaxman AD et al. *JAMA Ophthalmology.*

OK	18,700	13,900	24,100	0.48	0.35	0.61
OR	11,800	8,300	15,100	0.28	0.20	0.36
PA	52,300	35,700	68,500	0.41	0.28	0.54
RI	3,400	2,400	4,500	0.33	0.22	0.43
SC	20,900	15,900	26,300	0.42	0.32	0.52
SD	2,700	1,900	3,600	0.31	0.22	0.42
TN	28,500	21,400	35,800	0.42	0.32	0.53
TX	86,100	66,200	106,200	0.30	0.23	0.38
UT	5,900	4,500	7,700	0.19	0.14	0.25
VA	25,800	19,700	32,700	0.30	0.23	0.39
VT	2,400	1,600	3,100	0.38	0.26	0.49
WA	21,300	16,000	27,200	0.29	0.22	0.37
WI	16,600	11,800	21,800	0.29	0.20	0.38
WV	11,700	8,300	15,100	0.65	0.46	0.83
WY	1,600	1,100	2,000	0.27	0.20	0.35

© 2021 Flaxman AD et al. *JAMA Ophthalmology.*
