# Supplementary material for: Prevalence of Visual Acuity Loss or Blindness in the US: A Bayesian Meta-analysis
Source: JAMA Ophthalmol. 2021 May 13;139(7):717–23. doi: 10.1001/jamaophthalmol.2021.0527 (PMC8120442; doi:10.1001/jamaophthalmol.2021.0527)
Supplement: Supplement 2. — Nonauthor Collaborators. Vision and Eye Health Surveillance System Study Group members. [file jamaophthalmol-e210527-s002.pdf]

Group Name(s): Vision and Eye Health Surveillance System Study Group							
**First Name and Middle Initial(s)**	**Last Name**	**Suffix (eg, Jr, III)**	Academic Degrees	Institution	Location (city, state/province, country)	Role or Contribution, eg, chair, principal investigator	Group (if more than 1 Group listed in the byline) and/or Subgroup (eg, Steering Committee)
Kira N.	Baldonado						
Clare	Davidson						
Michelle C.	Dougherty						
Michael R.	Duenas						
David S.	Friedman						
Kevin M.	Jackson						
Charlotte E.	Joslin						
Barbara E.K.	Klein						
Phoebe A.	Lamuda						
Yao	Liu						
Flora C.	Lum						
Nnenna L.	Okeke						
Nita P.	Sinha						
Bonnielin K.	Swenor						
Jeff P.	Todd						
Emily	Tolbert						
